# Proteomic profile of tepary bean seed storage proteins in germination with low water potential

**DOI:** 10.1186/s12953-023-00225-6

**Published:** 2024-01-09

**Authors:** Daniel Padilla-Chacón, Laura Campos-Patiño, Cecilia B. Peña-Valdivia, Antonio García-Esteva, José Cruz Jiménez-Galindo, Jorge Luis Pizeno-García

**Affiliations:** 1 Colegio de Postgraduados, CONAHCYT-Programa de Posgrado en Botánica, Carretera México- Texcoco, km 36.5, Montecillo, 56264 México; 2 Programa de Posgrado en Botánica, Colegio de Postgraduados, Carretera México-Texcoco, km 36.5, Montecillo, 56264 México; 3 Campo Experimental Sierra de Chihuahua-INIFAP, Ciudad Cuauhtémoc, 31500 México

**Keywords:** Tepary bean, Germination, Seed storage protein, Low water potential, Proteomics

## Abstract

**Background:**

Tepary bean (*Phaseolus acutifolius* A. Gray) is one of the five species domesticated from the genus *Phaseolus* with genetic resistance to biotic and abiotic stress. To understand the mechanisms underlying drought responses in seed storage proteins germinated on water and polyethylene glycol (PEG-6000) at -0.49 MPa, we used a proteomics approach to identify potential molecular target proteins associated with the low water potential stress response.

**Methods:**

Storage proteins from cotyledons of Tepary bean seeds germinated at 24, 48 and 72 h on water and PEG-6000 at -0.49 MPa were analyzed by one-dimensional electrophoresis (DE) with 2-DE analysis and shotgun mass spectrometry. Using computational database searching and bioinformatics analyses, we performed Gene Ontology (GO) and protein interactome (functional protein association network) String analyses.

**Results:**

Comparative analysis showed that the effect of PEG-6000 on root growth was parallel to that on germination. Based on the SDS‒PAGE protein banding patterns and 2-DE analysis, ten differentially abundant seed storage proteins showed changes in storage proteins, principally in the phaseolin and lectin fractions. We found many proteins that are recognized as drought stress-responsive proteins, and several of them are predicted to be intrinsically related to abiotic stress. The shotgun analysis searched against UniProt’s legume database, and Gene Ontology (GO) analysis indicated that most of the seed proteins were cytosolic, with catalytic activity and associated with carbohydrate metabolism. The protein‒protein interaction networks from functional enrichment analysis showed that phytohemagglutinin interacts with proteins associated with the degradation of storage proteins in the cotyledons of common bean during germination.

**Conclusion:**

These findings suggest that Tepary bean seed proteins provide valuable information with the potential to be used in genetic improvement and are part of the drought stress response, making our approach a potentially useful strategy for discovering novel drought-responsive proteins in other plant models.

**Supplementary Information:**

The online version contains supplementary material available at 10.1186/s12953-023-00225-6.

## Background

The Tepary bean (*Phaseolus acutifolius* A. Gray) is part of the tertiary gene pool of the common bean produced in the Mexican western states and southwestern United States [1]. For many decades, Tepary bean has gained attention as a key source of genetic traits such as resistance to high and low temperatures [2, 3], salinity [4], and drought [5–10]. Although most studies have focused on seedling to mature plant physiological traits, minimal attention has been given to proteins that are directly involved in germination stress. Pioneering studies on the identification of Tepary bean proteins and mineral composition were conducted by electrophoresis [11–13]. However, over the last 20 years, there has been increasing interest in using mass spectrometry for proteins to elucidate the relationship between proteins and specific molecular interactions [14]. Several reports on the use of Shotgun proteomics have been conducted to study complex peptide fractions generated after protein proteolytic digestion in plants, including leaves of common bean [15] and seeds of barley [16], pea [17], *Brassica* [18], and quinoa [19]. However, some of the recent advances in the proteomics field, such as shotgun proteomics, have yet to be explored in species used in improving abiotic stress tolerance.

Tepary bean seeds have nutrimental parameters evaluated in the general range for legumes and other bean cultivars, with a relatively high percentage of protein (23–25%) and similarities to common bean [20]. The phaseolin fraction usually represents the largest proportion of proteins in grain and legumes (36–46%), with those inside the beans being the most abundant. Furthermore, lectins are 120 KDa tetramers (subunits approximately 31 KDa) and represent 5–12% of the total protein in the genus *Phaseolus* [21].

The objective of this study, through the application of PEG-6000, which cannot penetrate plant cell wall pores and simulates drought conditions in a short-term experiment, was to identify changes in storage proteins in cotyledons during seed germination and postgermination [22]. We have focused primarily to investigate the effect of osmotic stress simulated with PEG-6000 in Tepary bean cultivar (Tepary café) which have high tolerance to drought stress at -0.49 MPa [23] to generate knowledge in a basic understanding about of the changes in proteins associated to water deficit during the germination.

## Materials and methods

### Plant material and germination experiments

Seeds of the Tepary bean cv. Tepary café (*P. acutifolius* A. Gray) were used in this study. The material was collected in Sonora, Mexico, and was registered in the germplasm bank catalog for *Phaseolus* spp. of the National Institute of Forestry, Agriculture and Livestock Research (INIFAP) on Cuauhtémoc, Chihuahua, Mexico. Seeds were germinated in Petri dishes with filter paper at 25 ± 1 °C in the dark in a growth chamber. Two treatments were evaluated: one was the control, which included seed germination in distilled water, and the second was seed germination in 20% (w/v) PEG-6000 (ψ_W_ of -0.49 MPa) for 24, 48 and 72 h. This PEG-6000 concentration (-0.49 MPa) was chosen based on previous data obtained from germination percentage of common bean and Tepary bean [23]. The germination percentage (*n* = 70), the length of the radicle (*n* = 15), and the fresh weight (FW) were recorded. At least 2 mm of exposed radicle was the criterion for successful germination as described by the International Seed Testing Association [24].

### One dimensional electrophoresis

The embryonic axis-proximal seed structures (0.30–0.35 g) were excised from geminating seeds after 24, 48 and 72 h of the control and stressed treatments. Tissue was ground on ice for 1 min in 300 µL of buffer consisting of 2% (w/v) SDS, 0.1 M Tris/HCl (pH 7.6), 0.1 M dithiothreitol and 1% (v/v) Protease Inhibitor Cocktail (Sigma‒Aldrich). Then, the crude extract was centrifuged at 13,000 × *g* for 10 min at 4 °C, and the supernatant was collected. The protein concentration was measured with Bradford reagent using bovine serum albumin as a standard. Samples for electrophoresis were mixed with denaturing buffer, and the proteins were separated by SDS‒PAGE.

### Phenol protein extraction and 2-DE analysis

To demonstrate the quality of the 2-DE analysis and obtain a large number of spots, 1 g of cotyledons from seed cotyledons germinated at 72 h water (control) and − 0.49 MPa were placed in 6 mL of phenol pH 8.8 and 5 mL of extraction buffer (100 mM Tris HCl, pH 8.8, 10 mM EDTA, 900 mM sucrose and 0.4% 2-betamercaptoethanol). Total protein was extracted and separated by two-dimensional electrophoresis following the methods previously described by [25] with slight modifications.

### Identification of proteins by mass spectrometry

Identification of proteins by mass spectrometry samples was processed as described previously by [26]. The sample treatment prior to identification was carried out according to the protocol developed in the Research and Industry Support Services Unit (USAII) of the School of Chemistry at the National Autonomous University of Mexico (UNAM). Data processing and protein identification were performed with the global Protein Lynx version 2.4 server and software with a Protein Lynx Global Server (PLGS; Waters Corporation). PLGS scores with confidence > 95% were accepted as correct. The UniProt database was searched, and the peptides were matched with the theoretical peptides of the proteins reported for the samples.

### Annotations and gene ontology analysis

Gene ontologies (GOs) for the categories were obtained through the UniRef database. The CateGOrizer tool was used to identify the major GO categories of biological process, molecular function, and cellular compartment, generating bar graphs (http://www.animalgenome.org/cgi-bin/util/gotreei).

### Protein interactions

Protein interactomes (functional protein association networks) were constructed using the Multiple Protein by Name section of the String Database Version 12. Investigations of the interaction between phytohemagglutinin from *P. vulgaris* and other pathways in biological systems using String. Using the Multiple Protein by Name section of the String database, functional enrichment of the network was constructed based on the biological process using the data of counting proteins to create interactomes, which showed a cluster of proteins associated with protein folding.

### Statistical analyses

Statistical analyses were conducted using GraphPad Prism 10.02 (GraphPad Software, La Jolla, CA, United States). Statistically significant differences between control and osmotic stress are indicated: *p\0.05, **p\0.01, ***p\0.001 (ANOVA).

## Results

### Seed germination and seedling root growth

**Fig. 1:**
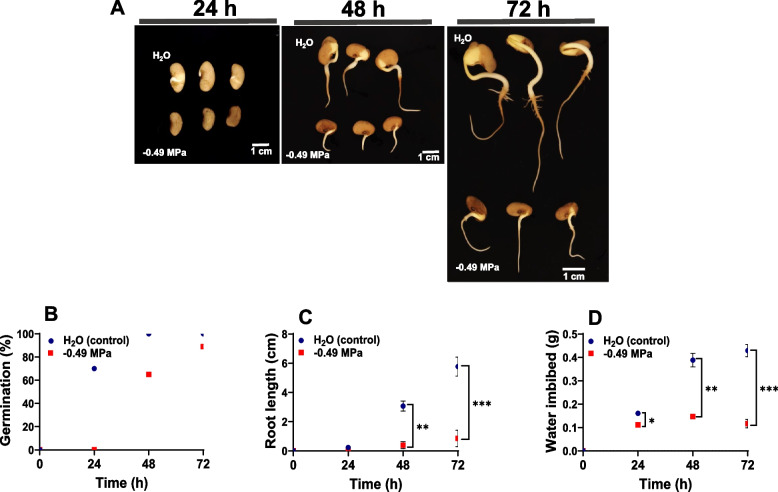
**A** Growth conditions and the course of the experiment from Tepary bean seeds (*Phaseolus acutifolius*) germinated at 24, 48, 72 h under water (control) and − 0.49 MPa. **B** Germination, **C** Root length and **D** Water imbibition. Statistically significant differences between control and osmotic stress were indicated: *p\0.05, **p\0.01, ***p\0.001 (ANOVA)

Seed germination in water reached a maximum after 48 h; in contrast, at -0.49 MPa, seeds took 72 h to reach 85% germination (Fig. 1A and B). Interestingly, at -0.49 MPa the root length displayed a non-significant increase between 48 and 72 h of germination while in water condition (control) was while in water condition (control) times higher (Fig. 1C). Seed imbibition was also contrasted between treatments. Imbibition represented increases between 0.15 and 0.4 g of fresh seed biomass after 24 to 48 h of germination in the control. In contrast, at -0.49 MPa, no significant seed fresh weight increase (*p* > 0.05) between 0.11 and 0.14 g was observed between 24 and 72 h (Fig. 1D).

### SDS‒PAGE and protein content

**Fig. 2:**
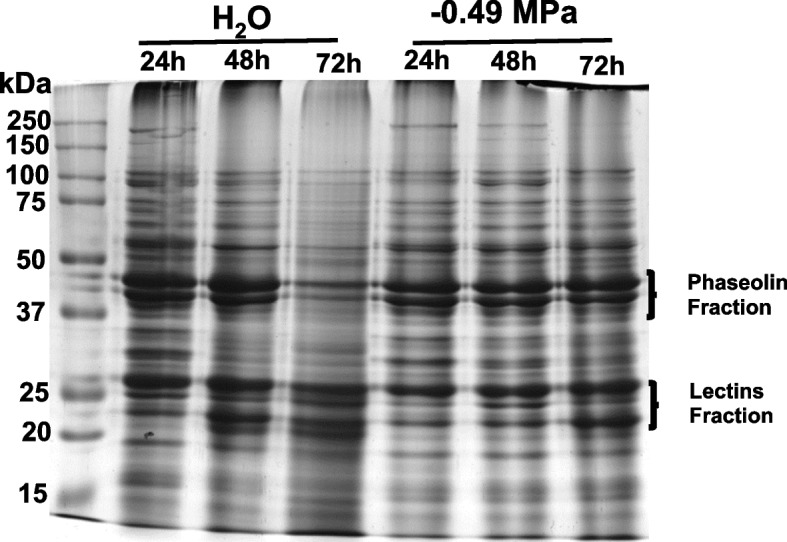
SDS-PAGE of extracted protein from Tepary bean cotyledons (*Phaseolus acutifolius*) germinated at 24, 48, 72 h under water (control) and − 0.49 MPa

The first aim of this study was to examine the protein profile by one-dimensional SDS‒PAGE using extracts of 24, 48 and 72 h seed germination in water and at -0.49 MPa in PEG-6000. Predominant protein bands with molecular weights between 20 and 25 kDa (lectin subunit fraction) and 50 kDa (phaseolin fraction) were present at 24, 48 and 72 h of imbibition (Fig. 2). However, the conspicuous phaseolin band of 45 kDa at 24 h of germination was abundant in water and decreased gradually between 48 and 72 h of imbibition, while at -0.49 MPa, the same band was diminished at all times. Similar protein patterns were observed in proteins with molecular weights of 25–30 kDa (Fig. 2). Interestingly, the lower band was increased in both treatments, and an additional band was detected in water at 72 h of imbibition but not at -0.49 MPa.

### Two-dimensional gel electrophoresis (2-DE)

**Fig. 3:**
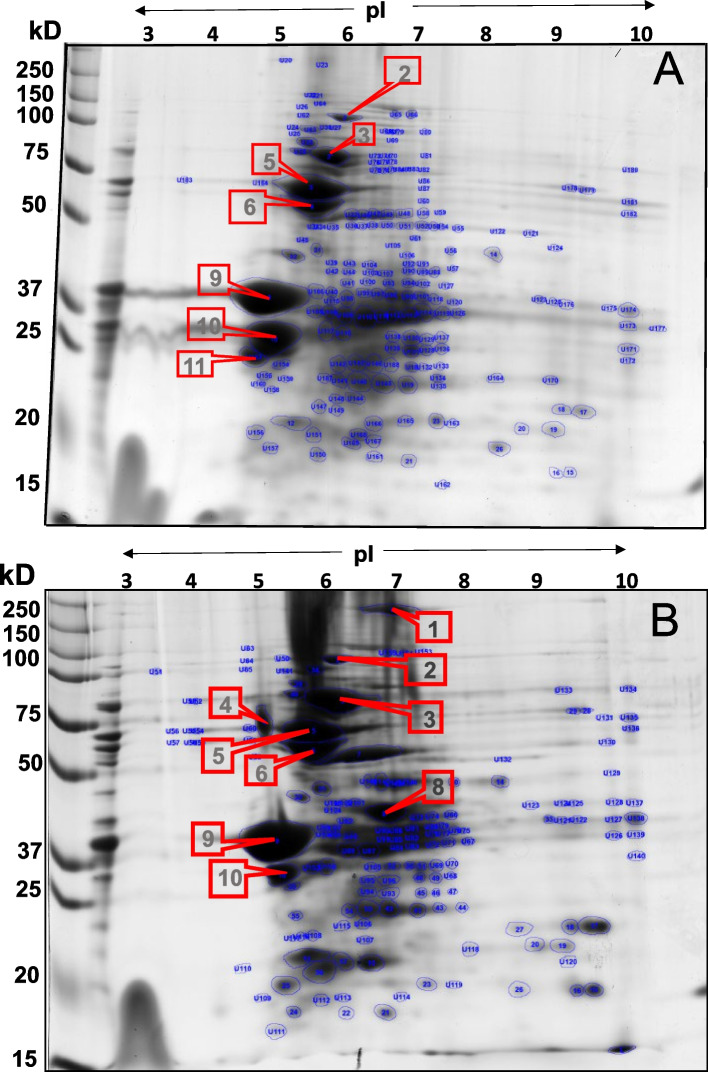
2-DE maps of *Phaseolus acutifolius* cotyledons from Tepary bean seeds with isoelectric points in the range of pH 3–10. **A** Water, **B** -0.49 MPa by 72 h of imbibition. Detailed information about the protein properties and identification of spot number coincidence is listed in Supplementary Data S1

To increase the profile of separation and identification of proteins, we performed a protein extraction method by phenol with extracts of seed cotyledons germinated at 72 h in water and − 0.49 MPa. This time point was chosen because it was the stage of germination with clear differences between stresses with − 0.49 MPa and the control. 2-DE analysis revealed marked differences in protein profiles (Fig. 3). The results showed a spatial distribution of the matched spots along the 2-DE gel with differences among the water and − 0.49 MPa treatments. The analysis by densitometry allowed us to generate a total number of 189 spots in 2-DE gels under water conditions and 151 spots in -0.49 MPa gels. The major proteins were identified with pI values in the 4–7 range. A second group of proteins was observed in the alkaline pI range, with more than 50 proteins with a pI of 8.5–9. Ten abundant storage proteins were identified in common among the water and − 0.49 MPa treatments, with significant differences in area obtained by densitometry (Supplementary Data S1) (Fig. 3A and B). Interestingly, one spot was only identified in water with a molecular weight of 23 kDa, while three spots with molecular weights of 250 kDa, 30 kDa and 75 kDa were identified only at -0.49 MPa (Fig. 3).

### Shotgun proteomics

To dissect the underlying mechanisms of water deficit in Tepary bean seeds, changes in protein profiles were explored by shotgun proteomics and identified with the UniProtk database, representing an “OK” value equal to 2. Based on sequence entries from H_2_O conditions, 16 annotations were identified for *P. vulgaris*, 3 for *P. acutifolius*, 2 for *Phaseolus angularis*, 2 for *Vigna radiata*, and 2 for *Vigna angularis* (Supplementary Data S2.1). In addition, sequence entries from PEG-6000 condition 27 were for *P. vulgaris*, 4 for *P. acutifolius*, 5 for *P. angularis*, 5 for *Vigna radiata*, 3 for *Vigna angularis* and 1 for *Rhizobium grahamii* (Supplementary Data S2.2).

**Fig. 4:**
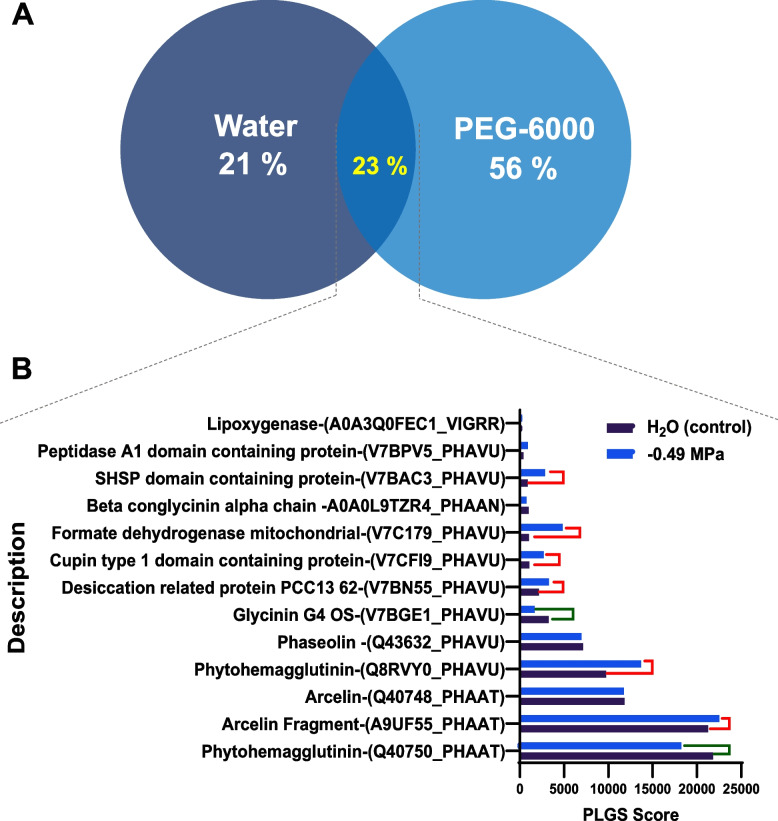
**A** Venn diagram of total differentially accumulated proteins from Tepary bean seeds obtained by shotgun proteomic analysis. Water (dark blue circle), -0.49 MPa (light blue circle) and **B** The overlap of total differentially accumulated proteins represented by PLGS score, calculated by Protein Lynx Global Serve, which higher score (Ok = 2) implies greater confidence of protein identity up-accumulated proteins and down-accumulated proteins from comparisons between water and − 0.49 MPa stress. The red and green lines implied protein up/downregulation observed respectively

Using the comparative proteomics analysis between water and -0.49 MPa, all accessions were grouped into 12 unique proteins for water (21 %), 32 for -0.49 MPa condition (56 %) and 13 for common proteins (23 %) (Fig. 4A). Interestingly, the results showed that the proteins phytohemagglutinin OS *Phaseolus acutifolius* OX and glycinin G4 OS *P. vulgaris* were significantly downregulated at -0.49 MPa with respect to water. In contrast, phytohemagglutinin OS *Phaseolus vulgaris* OX 3885, desiccation-related protein PCC13 62 OS *P. vulgaris*, cupin type 1 domain-containing protein OS *P. vulgaris*, formate dehydrogenase mitochondrial OS *P. vulgaris*, Arcelin Fragment and SHSP domain-containing protein OS *Phaseolus vulgaris* were upregulated with respect to water (Fig. 4B).

### Gene ontology

**Fig. 5:**
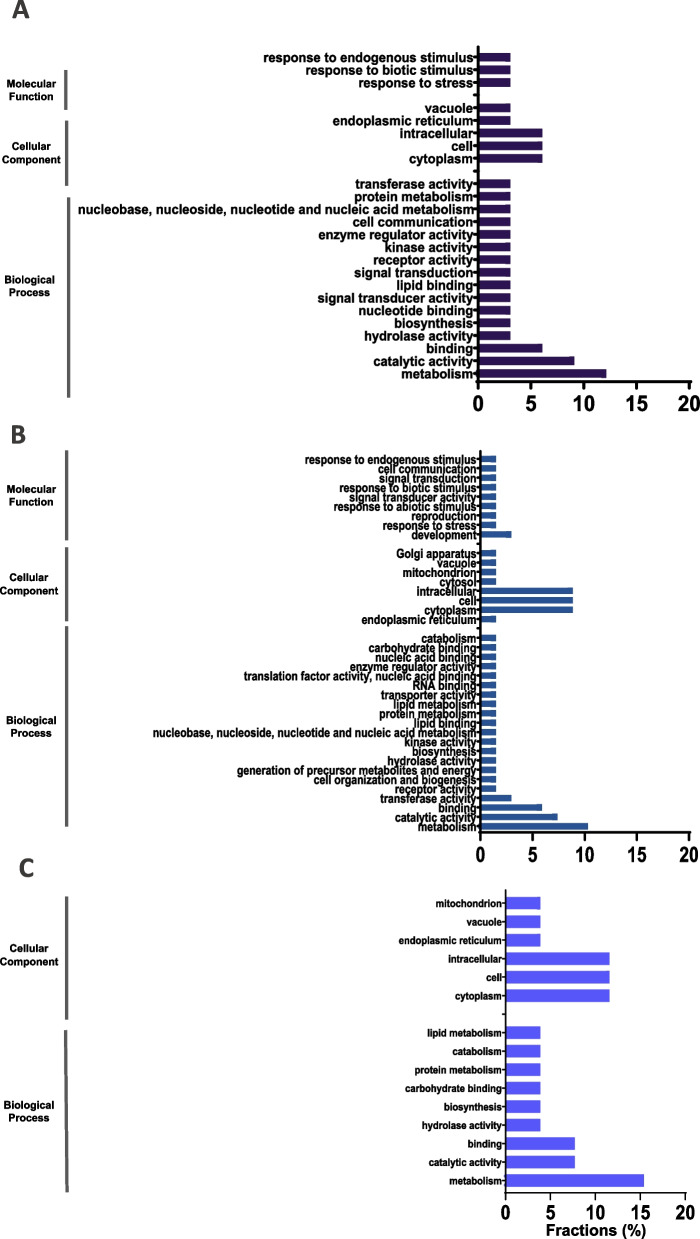
GO functional classification of definitions. GO terms are grouped into 3 ontologies: biological process, cellular component and molecular function. The analysis was based on unique protein accessions obtained from seeds germinated in **A** Water, **B** PEG-6000 (-0.49 MPa) and **C** Proteins in common

All proteins were classified in a careful analysis based on Gene Ontology (GO). The analysis was based on unique protein accessions obtained from seeds germinated in water, PEG-6000 and for proteins in common (Fig. 5). For water conditions, 20 unique terms from the dataset were found to belong to at least one of the 27 “GO_slim” classes (Supplementary Data S3.1). In conjunction with GO slim terms obtained from PEG-6000, a large number of GO terms were associated with biological processes compared with water; among these, 37 unique terms were found to belong to at least one of the 41 “GO_slim” classes (Supplementary Data S3.2). Finally, 12 unique terms were associated with at least one of the 18 “GO_slim” classes (Supplementary Data S3.3).

Among the proteins identified, GO terms in PEG-6000 were involved in many binding processes, such as carbohydrate binding, nucleic acid binding, lipid binding and RNA binding, and 40 proteins were involved in catabolism, protein metabolism, and kinase activity (Fig. 5).

The analysis was submitted to the STRING Database platform, which showed a cluster of proteins associated with legume lectin and the NAC domain (observed gene count = 6, strength = 2.03, false discovery rate = 4.64E-08), legume lectin and the NAC domain (observed gene count = 5, strength = 2, false discovery rate = 2.75E-06), legume lectin and epidermal patterning factor proteins (observed gene count = 4, strength = 2.38, false discovery rate = 3.78E-06), legume lectin and cellular manganese ion homeostasis (observed gene count = 3, strength = 2.52, false discovery rate = 0.00016), endocytic vesicle membrane and AT-hook motif nucleus-localized protein 15–29 (observed gene count = 2, strength = 3.01, false discovery rate = 0.0032), and legume lectin and cellular manganese ion homeostasis (observed gene count = 2, strength = 2.59, false discovery rate = 0.0133) (Table 1).

**Table 1:**
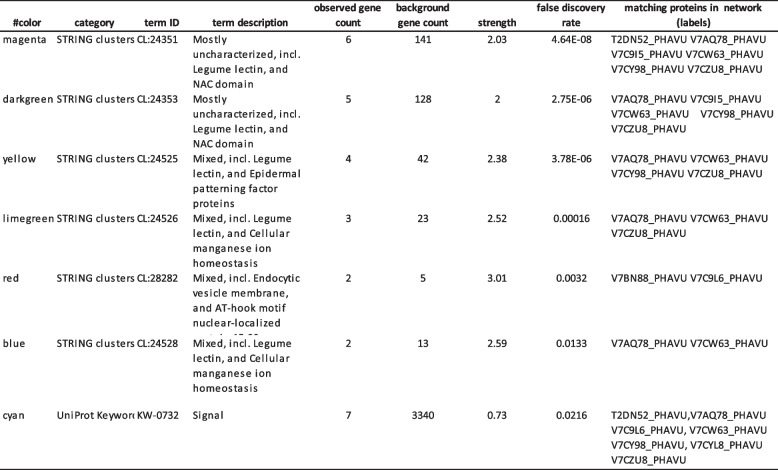
String analysis of seven proteins that interacted with Phytohemagglutinin from *P. vulgaris*

## Discussion

In the field, sowing time is selected based on climate and season, synchronous seed formation, and maturation before harvest, which is the basic requirement for increasing crop yield and quality [27]. Low seed water uptake is one factor that negatively affects common bean establishment in the field and leads to reduced seed germination and plant growth [28]. During seed germination of legumes, storage proteins, particularly in cotyledons, are hydrolyzed and used for new protein synthesis of other nitrogen-containing compounds [29]. Thus, the soil conditions that lead to either good or poor crop emergence depend on the environment and biotic or abiotic stressors, which can be of sufficient magnitude to affect water uptake by seeds. The close phylogenetic relationship between *P. vulgaris* and *P. acutifolius* enables Tepary bean to serve as a key source for drought tolerance. Previous reports indicated that during germination, seeds of *P. vulgaris* are more sensitive to low water potential than *P*. *acutifolius* [23], even with the highly drought-tolerant cultivation of common beans [30, 31]. However, the regulatory pathway that confers an active mechanism of protein degradation under water restriction is not well understood. This study showed that at -0.49 MPa, seed germination was delayed by 24 h, and root length decreased by 80% in comparison to the control (Fig. 1B). Interestingly, seed imbibition reached a maximum when roots emerged and was maintained at a constant level (Fig. 1D). This suggests that even under low water availability, seed reserves are used to maximum during growth under stress.

In this work, protein profiling studies in Tepary were conducted to demonstrate the changes in protein presence and abundance during seed germination at 24, 48, and 72 h. The SDS‒PAGE gel mainly showed changes in smaller bands of 15–20 kDa and 110 (high molecular weight) kDa during 48–72 h of germination (Fig. 2). Similarly, the phaseolin fraction (50 kDa) decreased quickly after 24 h in water conditions and less accelerated at a low water potential of -0.49 MPa (Fig. 2). Although phaseolin is not directly related to drought stress in the seeds of common bean, the abundance of *P. acutifolius* decreased in both treatments. Is well knowed that phaseolin fraction is the major seed storage protein in cotyledon of Tepary bean [21]. However, the regulatory mechanisms responsible for the synthesis, accumulation and degradation of phaseolin in the common bean seed are not yet sufficiently known [32–34]. Further investigation will be done in our future to understand why the mayor storage of protein manifiest resistence to proteolityc degradation under − 0.49 MPa. In contrast, the 20–25 kDa lectin protein fraction appeared to have changed with an additional band observed after 72 h of germination in water, while at -0.49 MPa, the band was absent (Fig. 2).

To increase the resolution and number of proteins in Tepary bean seeds, the phenol method for protein extraction was used to produce a 2-DE gel [35]. Among all the proteins observed, ten proteins showed the most significant changes in abundance during seed germination in water and at -0.49 MPa in PEG-6000 (Fig. 3A and B) (Supplementary Data S1). Based on the spot coincidence on the experimental gels, a significant difference in fold change can be suggested, particularly due to the absence of spot number 11 (Fig. 3B) when at low water potential, which, according to MW and pI, represents a lectin fraction. Many different studies have been reported on common bean seed proteome analysis using 2-DE coupling [15]. In addition, our data from 2-DE agreed with previous findings with a comparative 2-DE analysis undertaken in common bean seed, whereas six arcelin isoforms and one phytohemagglutinin isoform were altered in lines with deficiency and lack of phaseolin, phytohemagglutinin, and arcelin. Here, the results indicated that the changes in spot patterns in water and at -0.49 MPa in PEG-6000 may implicate protein degradation and synthesis due to proteolysis or posttranslational modifications and possible exploitation. Previous reports demonstrated that phytohemagglutinin (PHA) is a lectin extracted from *P. vulgaris* seeds that contains potent cell agglutinating capacity and mitogenic activities [36]. Additionally, several plant lectins have been recognized and functionally characterized [37]. There has been a resurgence of interest in exploring the role of lectins in modulating various biological responses in plants. In particular, the changes have been evaluated to determine if the expression of PHA genes along with changes in the DNA methylation patterns at the PHA locus are predominantly located in cotyledons of common bean [38]. Recently, lectins have been recognized in rice, and the R40 family of these proteins contains the carbohydrate-binding ricin-like domain and shows responses to osmotic stress [39]. That study revealed that mobilization of phaseolin in germinating seeds seems to occur through the degradation of highly phosphorylated isoforms. Therefore, the regulatory mechanisms responsible for the synthesis and subsequent degradation of phaseolin through phosphorylation might undergo variations in Tepary bean seeds.

Other seed proteins that showed changes among both treatments were small heat shock protein (SHSP), domain-containing protein V7BAC3_PHAVU, and formate dehydrogenase mitochondrial V7C179_PHAVU, which showed high PLGS scores in PEG-6000 in comparison with water (Fig. 4). In plants, as in other organisms, sHSPs are upregulated by stress and are proposed to act as molecular chaperones to protect other proteins from stress-induced damage [40], and formate dehydrogenase (FDH, EC 1.2.1.2.) which is a mitochondrial, NAD-dependent enzyme reported in potato (*Solanum tuberosum* L.) and is involved in formate-dependent O_2_ uptake coupled to ATP synthesis in green leaves [41]. These findings thus revealed that these proteins are involved in low water potential during germination. However, their involvement in the components of machinery stress tolerance has yet to be analyzed or comprehended in greater detail.

Identified seed proteins were classified by Gene Ontology (GO) terms in three broad domains – biological process, cellular component, and molecular function – for each group of proteins (Fig. 5).

The majority of biological process proteins induced by PEG-6000 were associated with carbohydrate binding, nucleic acid binding, lipid binding, RNA binding, catabolism and protein metabolism. These findings suggest that the response to stress involves rapid reprogramming of gene expression, which impacts the proteome and cellular metabolism. In this regard, posttranscriptional gene regulation largely relies on RNA-binding proteins (RBPs), which recognize and bind to specific target RNAs to modulate the activity and fate of RNA transcripts [42]. According to carbohydrate binding, lectins have diverse functions in cell signaling associated with growth and development, as well as in response to biotic, symbiotic, and abiotic stimuli, which is attributed to the differences in the amount of information available for some of the well-characterized proteins with detailed annotations [43].

**Fig. 6:**
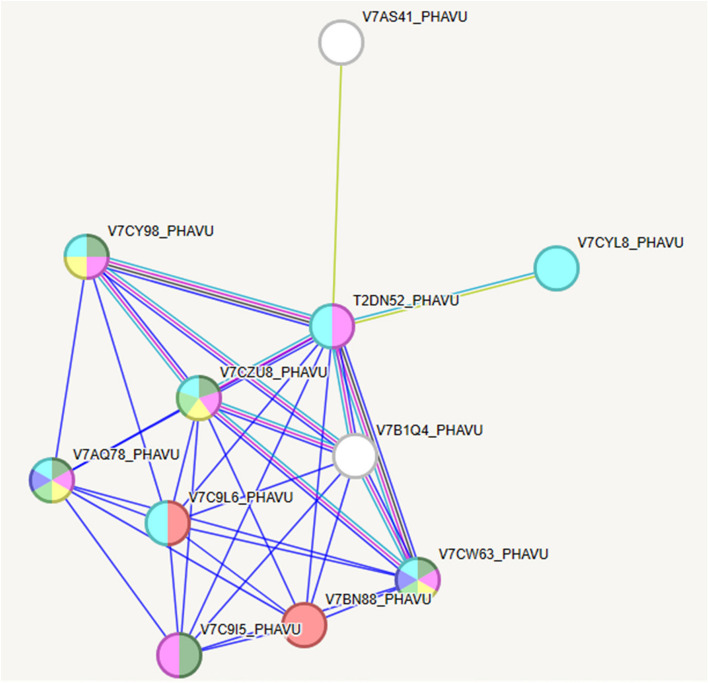
Analysis of a functional network by STRING 12.0 (http://string-db.org). *Phaseolus vulgaris* and Phytohemaglutinnin were used for analysis parameters with *P. vulgaris* organism. Ten clusters of highly interacting protein nodes are marked with circles and include proteins involved in the network. Different line colors represent the types of evidence used in predicting the associations (Table 1)

To further reduce this complexity and provide an easy visual of the major GO terms associated with the seed proteome, the String database was used to predict physical and functional protein‒protein interactions. In this work, we selected phytohemagglutinin from *P. vulgaris* (Fig. 6) to describe the interaction network functional enrichment analysis on the interaction between phytohemagglutinin and other pathways using the String database. The results provided evidence of interactions with 10 proteins (Fig. 6). Although it is unclear, the induction of Pept_C1 domain-containing protein belongs to the peptidase C1 family. Identifier: T2DN52 and T2DN52_PHAVU may be another mechanism favored by phytohemagglutinin to control osmotic stress (Table 1). Previous reports indicate that endopeptidase designated EP-C1 is expressed in the germinated cotyledons of common bean seeds [44], and proteinases play a significant role in the protein degradation in the cotyledons by the RY element in the promoters of the genes for protease and α-amylase, which were expressed in germinated seeds of *Vigna mungo* [45].

These new details help us to understand the tolerance to water deficit, especially Tepary bean proteins, which may provide essential information to genetic improvement programs in common bean and are agronomically important because the speed and uniformity of seed germination and seedling emergence ensures early plant establishment and weed competition avoidance.

## Conclusion

Our proteomics analysis data on Tepary bean seeds germinated under low water potential demonstrate that lectins may play an important role in germination by upregulating storage protein degradation before root emergence. Therefore, we speculate that phytohemagglutinin acts as a modulator with additional proteins that should improve germination at low water potential, which could be used for genetic improvement.

### Supplementary Information

**Additional file 1: Supplementary Data S1.** Changes in accumulation of ten differential protein spots from Tepary bean seeds under water (control) and low water potential with PEG-6000 at -0.49 MPa after 72h germination. ND=No Detected. **Supplementary Data S2.1.** Proteins Identified by Shotgun proteomics from cotyledon of Tepary bean seeds germinated in water during 72h. **Supplementary Data S2. 2.** Proteins Identified by Shotgun proteomics from cotyledon of Tepary bean seeds germinated with low water potential at -0.49 MPa during 72h. **Supplementary Data S3.1.** Mapping of 27 GO terms to 127 of the ¨GO slim¨ from proteins of cotyledons of Tepary bean seeds germinated in water during 72 h and identified by Shotgun Proteomics UniProt. **Supplementary Data S3.2.** Mapping of 72 GO terms to 127 of the ¨GO slim¨ from proteins of cotyledons of Tepary bean seeds germinated in PEG-6000 at -0.49 MPa during 72 h and identified by Shotgun Proteomics UniProt. **Supplementary Data S3.3.** Mapping of 18 GO terms to 127 of the ¨GO slim¨ from common proteins of cotyledons of Tepary bean seeds germinated in water and PEG-6000 at-0.49 MPa during 72 h and identified by Shotgun Proteomics UniProt.

## Data Availability

All data supporting this study were included in article.

## Abbreviations

MPa: Mega Pascal
KDa: Kilodalton
SDS‒PAGE: Sodium dodecyl sulfate polyacrylamide gel electrophoresis
EDTA: Ethylenediaminetetraacetic acid
