# Supplementary material for: Proteomic profile of tepary bean seed storage proteins in germination with low water potential
Source: Proteome Sci. 2024 Jan 9;22:1. doi: 10.1186/s12953-023-00225-6 (PMC10775562; doi:10.1186/s12953-023-00225-6)
Supplement: Supplementary file 1 — Additional file 1: Supplementary Data S1. Changes in accumulation of ten differential protein spots from Tepary bean seeds under water (control) and low water potential with PEG-6000 at -0.49 MPa after 72h germination. ND=No Detected. Supplementary Data S2.1. Proteins Identified by Shotgun proteomics from cotyledon of Tepary bean seeds germinated in water during 72h. Supplementary Data S2. 2. Proteins Identified by Shotgun proteomics from cotyledon of Tepary bean seeds germinated with low water potential at -0.49 MPa during 72h. Supplementary Data S3.1. Mapping of 27 GO terms to 127 of the ¨GO slim¨ from proteins of cotyledons of Tepary bean seeds germinated in water during 72 h and identified by Shotgun Proteomics UniProt. Supplementary Data S3.2. Mapping of 72 GO terms to 127 of the ¨GO slim¨ from proteins of cotyledons of Tepary bean seeds germinated in PEG-6000 at -0.49 MPa during 72 h and identified by Shotgun Proteomics UniProt. Supplementary Data S3.3. Mapping of 18 GO terms to 127 of the ¨GO slim¨ from common proteins of cotyledons of Tepary bean seeds germinated in water and PEG-6000 at-0.49 MPa during 72 h and identified by Shotgun Proteomics UniProt. [file 12953_2023_225_MOESM1_ESM.docx]

Supplementary Data S1. Changes in accumulation of ten differential protein spots from Tepary bean seeds under water (control) and low water potential with PEG-6000 at -0.49 MPa after 72h germination. ND=No Detected.

Supplementary Data S2.1. Proteins Identified by Shotgun proteomics from cotyledon of Tepary bean seeds germinated in water during 72h.

Supplementary Data S2.2. Proteins Identified by Shotgun proteomics from cotyledon of Tepary bean seeds germinated with low water potential at -0.49 MPa during 72h.

Supplementary Data S3.1. Mapping of 27 GO terms to 127 of the ¨GO slim¨ from proteins of cotyledons of Tepary bean seeds germinated in water during 72 h and identified by Shotgun Proteomics UniProt.

Go term used in the analysis obtained from cotyledons of seed germinated in water: GO:0036211, GO:0009987, GO:0003824, GO:0009719, GO:0000166, GO:0042221, GO:0009987, GO:0009058, GO:0008152, GO:0006950, GO:0006139, GO:0016301, GO:0462221, GO:0009607, GO:0007165, GO:0006950, GO:0038023, GO:0030234, GO:0008289, GO:0016787, GO:0042221, GO:0009987, GO:0003824, GO:0006950, GO:0005773, GO:0005783, GO:0016020

Supplementary Data S3.2. Mapping of 72 GO terms to 127 of the ¨GO slim¨ from proteins of cotyledons of Tepary bean seeds germinated in PEG-6000 at -0.49 MPa during 72 h and identified by Shotgun Proteomics UniProt.

**Go term used in the analysis obtained from cotyledons of seed germinated in PEG-6000 at -0.49 MPa.** GO:0005739,GO:0009987,GO:0009056,GO:0003824,GO:0030246,GO:0042221,GO:0036211,GO:0009628,GO:0009987,GO:0006950,GO:0003824,GO:0005783,GO:0005773,GO:0016020,GO:0003824,GO:0009987,GO:0009058,GO:0008152,GO:0006139,GO:0016301,GO:0042221,GO:0009987,GO:0003824,GO:0003824,GO:0009987,GO:0008152,GO:0006091,GO:0003824,GO:0005829,GO:0005794,GO:0016043,GO:0009987,GO:0006139,GO:0003824,GO:0009791,GO:0007275,GO:0000003,GO:0042221,GO:0009719,GO:0009607,GO:0007165,GO:0006950,GO:0038023,GO:0030234,GO:0016787,GO:0008289,GO:0005783,GO:0003824,GO:0030246,GO:0006091,GO:0016491,GO:0005215,GO:0009987,GO:0009058,GO:0008152,GO:0016740,GO:0005488,GO:0009987,GO:0008152.GO:0006091,GO:0003824,GO:0005783,GO:0005773,GO:0005737,GO:0008135,GO:0005737,GO:0009058,GO:0009987,GO:0009058,GO:0006629, GO:0005488,GO:0003824

Supplementary Data S3.3. Mapping of 18 GO terms to 127 of the ¨GO slim¨ from common proteins of cotyledons of Tepary bean seeds germinated in water and PEG-6000 at -0.49 MPa during 72 h and identified by Shotgun Proteomics UniProt.

**Go term used in the analysis obtained from common conditions of germination water and PEG-6000 at -0.49 MPa.** GO:0030246,GO:0030246,GO:0030246,GO:0030246,GO:0030246,GO:0005773,GO:0005739,GO:0005783,GO:0003824,GO:0009056,GO:0009987,GO:0019538,GO:000998,GO:0009058 GO:0016787,GO:0006629,GO:0005488,GO:0003824
